# Bearing Fault-Detection Method Based on Improved Grey Wolf Algorithm to Optimize Parameters of Multistable Stochastic Resonance

**DOI:** 10.3390/s23146529

**Published:** 2023-07-19

**Authors:** Weichao Huang, Ganggang Zhang

**Affiliations:** 1 Shannxi Key Laboratory of Complex System Control and Intelligent Information Processing, Xi’an University of Technology, Xi’an 710048, China; 2 School of Automation and Information Engineering, Xi’an University of Technology, Xi’an 710048, China; zgg@stu.xaut.edu.cn

**Keywords:** multistable stochastic resonance, adaptive parameter, improved grey wolf algorithm, bearing fault detection

## Abstract

In an effort to overcome the problem that the traditional stochastic resonance system cannot adjust the structural parameters adaptively in bearing fault-signal detection, this article proposes an adaptive-parameter bearing fault-detection method. First of all, the four strategies of Sobol sequence initialization, exponential convergence factor, adaptive position update, and Cauchy–Gaussian hybrid variation are used to improve the basic grey wolf optimization algorithm, which effectively improves the optimization performance of the algorithm. Then, based on the multistable stochastic resonance model, the structure parameters of the multistable stochastic resonance are optimized through improving the grey wolf algorithm, so as to enhance the fault signal and realize the effective detection of the bearing fault signal. Finally, the proposed bearing fault-detection method is used to analyze and diagnose two open-source bearing data sets, and comparative experiments are conducted with the optimization results of other improved algorithms. Meanwhile, the method proposed in this paper is used to diagnose the fault of the bearing in the lifting device of a single-crystal furnace. The experimental results show that the fault frequency of the inner ring of the first bearing data set diagnosed using the proposed method was 158 Hz, and the fault frequency of the outer ring of the second bearing data set diagnosed using the proposed method was 162 Hz. The fault-diagnosis results of the two bearings were equal to the results derived from the theory. Compared with the optimization results of other improved algorithms, the proposed method has a faster convergence speed and a higher output signal-to-noise ratio. At the same time, the fault frequency of the bearing of the lifting device of the single-crystal furnace was effectively diagnosed as 35 Hz, and the bearing fault signal was effectively detected.

## 1. Introduction

The failure rate of rolling bearings accounts for about 30% of all rotating machinery failures, which is the main reason affecting the operating efficiency, productivity, and life of mechanical equipment. Almost all rolling bearing fault signals are in a very noisy environment, resulting in early weak faults that are difficult to find. Therefore, how to enhance the signal-to-noise ratio of fault signals under extreme conditions has become a key issue in the direction of fault diagnosis. At the same time, monitoring the status of rolling bearings, promptly identifying faults, and conducting equipment maintenance are of great practical significance for ensuring the smooth working of rotating machinery systems [1]. Nowadays, the main methods used for rolling bearing fault detection are: wavelet decomposition [2], empirical mode decomposition [3], variational mode decomposition [4], principal component analysis [5], stochastic resonance [6], etc. The stochastic resonance algorithm overturns the view that noise is harmful for a long time. It uses the resonance principle to transfer noise energy to the fault signal, thus improving the detection and diagnosis of the fault signal, and opening up a new method and idea for weak bearing fault-signal detection submerged in strong noise.

Benzi raised the concept of stochastic resonance (SR) in 1981 when studying the changes of the Earth’s ice ages [7]. After 40 years of development, SR theory has been widely used in fault diagnosis [8], optics [9], medicine [10], image denoising [11], and other fields, and has achieved many remarkable results. The SR algorithm makes use of the synergy generated by the joint excitation of nonlinear systems, input signals, and noise to make Brownian particles oscillate, improve the output signal-to-noise ratio, and effectively detect the measured signal, which is a typical method to enhance the measured signal. Therefore, it is widely concerned with the domain of signal detection [12]. Classical bistable and monostable SR models have been extensively used in the study of signal detection [13]. However, for the signal to be measured with ultra-low amplitude, due to the potential function structure constraints, particles are often unable to effectively jump between potential wells, and SR-detection methods for bistable and monostable models are also powerless. When studying multistable stochastic resonance systems, Li et al. found that the multistable model can better enhance the output signal-to-noise ratio and improve the noise utilization ratio than the bistable and monostable models [14]. Therefore, more and more scholars have carried out relevant research on multistable SR [15]. For example, Zhang et al. proposed a piecewise unsaturated multistable SR (PUMSR) method which overcomes the weakness of tri-stable SR output saturation and enhances the ability of weak signal detection [16].

However, whether it is a monostable, bistable, or multistable SR algorithm, it is inevitably difficult to select model parameters in practical applications. Mitaim et al. [17] put forward the adaptive SR theory to enhance useful signals by automatically adjusting the structural parameters of nonlinear systems. But, the adaptive SR method, which takes a single parameter of the system as the optimization object, often ignores the interaction between the parameters of the system. With the rise of the swarm intelligence optimization algorithm, finding the global optimal solution through the swarm intelligence algorithm can solve the limitations of traditional adaptive SR systems, and this concept has been extensively used in the domain of bearing fault detection [18]. However, in the existing research results, the adaptive selection of SR model parameters still depends on the performance of intelligent optimization algorithms, so there are generally issues such as a low solving accuracy and being prone to falling into local optima [19]. Therefore, the feasible method to effectively enhance the parameter performance of adaptive selection of SR systems is to improve the defects of the intelligent optimization algorithm, so that it can more quickly and accurately optimize the parameters of the SR system. The grey wolf optimization algorithm can find the optimal solution by simulating the tracking, encircling, pursuit, and attack stages of the group predation behavior of the grey wolf. With few parameters and a simple structure, it is easy to integrate with other algorithms for improvement, but there are also the problems that it is easy to fall into local optimal solutions and low computational efficiency [20]. Therefore, it is of great research value to improve the basic grey wolf algorithm and improve its optimization performance [21]. Vasudha et al. proposed a multi-layer grey wolf optimization algorithm to further achieve an appropriate equivalence between exploration and development, thereby improving the efficiency of the algorithm [22]. Rajput et al. proposed an FH model based on the sparsity grey wolf optimization algorithm, which helps to minimize the computational overhead and improve the computational accuracy of the algorithm [23].

This article takes bearing fault-signal detection as the research object. Aiming at the problem of difficult parameter selection of multistable SR systems, a bearing fault-detection method based on an improved grey wolf algorithm to optimize multistable SR parameters is raised. This method improves the basic grey wolf optimization algorithm. Firstly, considering the quality of the initial solution, a Sobol-sequence initialization population strategy is proposed to make the distribution of the initial grey wolf population more uniform. Secondly, a convergence-factor adjustment strategy based on exponential rules is proposed to coordinate the global exploration and local development stages of the algorithm. Meanwhile, an adaptive position-update strategy is proposed to improve the accuracy of the algorithm, and Cauchy–Gaussian mixture mutation is used to enhance the algorithm’s ability to escape from local optima. Experimental verification is conducted on the performance of the improved grey wolf algorithm using fifteen benchmark test functions from the CEC23 group of commonly used test functions. The verification results display that the multi-strategy improved grey wolf optimization algorithm (MSGWO) has a faster convergence speed and a higher convergence accuracy. Then, on the basis of the model of the multistable SR system, the parameters of the multistable SR system are optimized through the MSGWO, so as to enhance the fault signal and realize the effective detection of the bearing fault signal. Finally, the bearing fault-detection method raised in this article is used to analyze and diagnose a bearing data set from Case Western Reserve University (CWRU) and a bearing data set from the Mechanical Fault Prevention Technology Association (MFPT), and is compared with the optimization results of other improved algorithms. Meanwhile, the method raised in this article is used to diagnose the fault of the bearing of the lifting device of a single-crystal furnace. The test results display that the bearing fault-detection method raised in this article has a fast convergence speed and a large output signal-to-noise ratio, and can detect bearing fault signals accurately and efficiently.

The rest of this article is arranged as below: The Section 2 introduces the specific cases of bearing failure in rotating machinery in different industries. The Section 3 introduces the basic principle of multistable SR. The Section 4 introduces the principle of the basic grey wolf optimization algorithm and the MSGWO, and compares it with some basic optimization algorithms and improved optimization algorithms, respectively. At the same time, the population diversity and the exploration and development stage of the MSGWO are analyzed. The Section 5 introduces the bearing fault-diagnosis method based on the MSGWO to optimize the multistable SR parameters, and uses the proposed method to analyze and diagnose the bearing data sets from CWRU and the MFPT. Meanwhile, the raised method is used to diagnose the bearing fault of the monocrystal furnace lifting device. The Section 6 is the summary.

## 2. Specific Cases of Bearing Failure

Due to the diverse working environments of bearings during the operation of rotating machinery, they are easily affected by wear, corrosion, and other factors, making it easy for various faults to occur. For example, in June 1992, during the overspeed test of a 600 MW supercritical active generator set at the Kansai Electric Power Company Hainan Power Plant in Japan, the bearing failure of the unit and the critical speed drop caused strong vibration of the unit, resulting in a crash accident and economic losses of up to JPY 5 billion. From September 2003 to October 2004, the China Railway Beijing–Shanghai Line, Shitai Line, and Hang-gan Line had a total of five traffic incidents. According to relevant statistics, four of these accidents were caused by train bearing-fatigue fracture, with a total economic loss of up to CNY 2 billion. In April 2015, China Dalian West Pacific Petrochemical Co., LTD., due to the serious distortion and fracture of the inner ring of the driving end bearing and the serious wear and deformation of the bearing ball, the seal of the bottom pump of the stripping tower of a hydrocracking unit quickly failed, and the medium leaked, which caused a fire. The accident caused three pumps, the frame above the pump, and a small number of meters and power cables to set fire; a local pipeline to crack; and direct economic losses of CNY 166,000. In 2018, the US Navy’s “Ford” aircraft carrier had to return to the shipyard for maintenance due to a thrust bearing failure during a mission. In August 2019, when a drone was spraying pesticides at a farm in Hebei, China, its motor rolling bearing failed, causing the drone to lose control, and a large amount of pesticides were spilled into the river, causing serious pollution. In December 2021, there were two recessive cracks in the bearing of unit #33 of a wind farm in Liaoning, China. Due to the limited installation position, the appearance inspection could not find them. As a result, the shaft cracks were promoted by the wind wheel’s alternating load during operation, resulting in a spindle fracture and the impeller’s overall fall. Therefore, the research on fault-diagnosis technology of rolling bearings is very necessary and has great practical significance.

## 3. Basic Principles of Multistable SR

### 3.1. The Basic Theory of Multistable SR

The principle of SR is that weak characteristic signals can be enhanced and detected by noise transfer mechanism under the action of nonlinear system. In general, when interpreting the SR model, we should first consider Langevin’s dynamic equation [24], which is as follows:

(1) $$\frac{d^{2}x}{dt^{2}}+\frac{dx}{dt}=-U^{\prime }(x)+s(t)+n(t)$$

where x is the system response of SR, U(x) is a class of nonlinear multistable potential function, s(t) is the external incentive, n(t) is the noise excitation, m is the mass of the particle, and k is the drag coefficient.

The definition formula of the nonlinear multistable potential function is:

(2) $$U(x)=\frac{a}{2}x^{2}-\frac{1+a}{4b}x^{4}+\frac{c}{6}x^{6}$$

**Figure 1:**
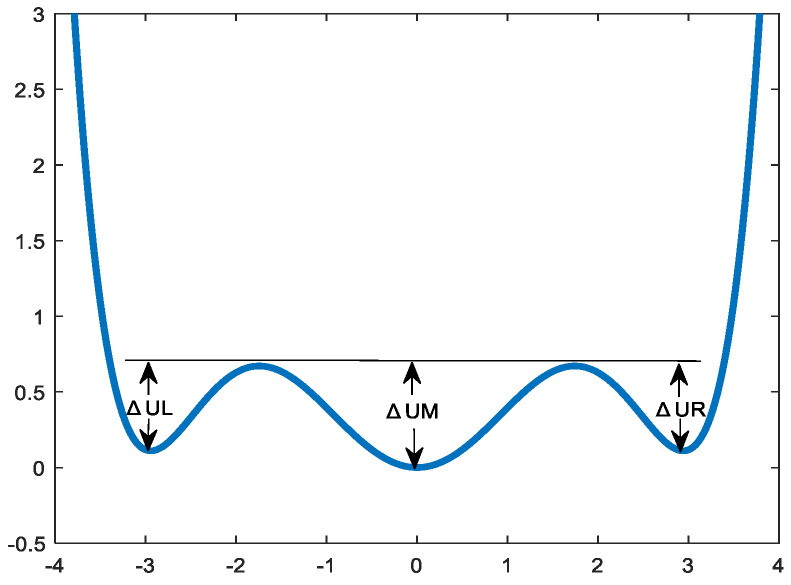
Potential function curve of multistable system.

In the formula, a, b, and c are parameters of the nonlinear multistable model, and they are all greater than 0. The potential function model image of the multistable system is displayed in Figure 1.

Substitute the potential function of the multistable model into Formula (1), add noise with intensity D in the system, and then obtain the Langevin equation of the nonlinear multistable system as follows:

(3) $$\frac{dx}{dt}=-ax+\frac{1+a}{b}x^{3}-cx^{5}+s(t)+\sqrt{2D}n(t)$$

When periodic signal and noise signal are used as excitation simultaneously, the inclination of potential well in the multistable system will increase. In addition, the periodic signal will also make the potential well depth of the three potential wells of the multistable potential function change periodically, and can guide the noise signal to switch synchronously. When the signal, noise, and multistable SR system reach a certain matching relationship, particles can make periodic transitions between potential wells, so that the components of the system output with the same frequency as the input signal are strengthened.

### 3.2. System Parameters’ Range

The fourth order Runge–Kutta formula was used to solve the multistable SR model. The specific calculation formula is:

(4) $$\left\{ \begin{array}{l} k_{1}=h(-ax(n)+\frac{1+a}{b}x^{3}(n)-cx^{5}(n)+s(n))\\ k_{2}=h(-a(x(n)+\frac{k_{1}}{2})+\frac{1+a}{b}{\left(x(n)+\frac{k_{1}}{2}\right)}^{3}-c{\left(x(n)+\frac{k_{1}}{2}\right)}^{5}+s(n))\\ k_{3}=h(-a(x(n)+\frac{k_{2}}{2})+\frac{1+a}{b}{\left(x(n)+\frac{k_{2}}{2}\right)}^{3}-c{\left(x(n)+\frac{k_{2}}{2}\right)}^{5}+s(n))\\ k_{4}=h(-a(x(n)+k_{3})+\frac{1+a}{b}{\left(x(n)+k_{3}\right)}^{3}-c{\left(x(n)+k_{3}\right)}^{5}+s(n))\\ x(n+1)=x(n)+\frac{1}{6}(k_{1}+2k_{2}+2k_{3}+k_{4}) \end{array}\right.$$

where x(n) is the nth sampling value of the system output, s(n) is the nth sampling value of the noise-added input signal, h is the sampling step, and $k_{i}\left(i=1,2,3,4\right)$ is the slope of the output response at the relevant integration point.

Normally, due to noise, particles jump over higher barrier heights by accumulating energy, so b, c, and h take the real numbers of [0, 10]. As the target signal is relatively weak, the interval in [25] is quoted; the range of a is set to [0, 0.5].

## 4. Multi-Strategy Improved Grey Wolf Optimization Algorithm

### 4.1. The Primary Theory of Grey Wolf Optimization Algorithm

Grey Wolf Optimizer (GWO) is a new intelligent swarm optimization algorithm proposed by Mirjalili et al. [26], whose main ideas are the leadership hierarchy and group hunting mode of grey wolf groups. The grey wolf population has a strict hierarchy. The head of the population is α, which represents the most coordinated individual in the wolf pack, and is mainly responsible for the decision-making affairs of the group’s predation behavior. The β wolf is second only to α in the population, and its role is to serve the α wolf to make decisions and deal with the behavior of the population. The third rank in the population is the δ wolf, which obeys the instructions issued by the α and β, but has command over other bottom individuals. The lowest individual in the group, known as ω, is submissive to the instructions of other higher-ranking wolves and is primarily responsible for balancing the relationships within the group. GWO defines the three solutions with the best fitness as α, β, and δ, while the remaining solutions are defined as ω. The hunting process (optimization process) is guided by α, β, and δ to track and hunt the prey (position update), and finally complete the hunting process, that is, obtain the optimal solution. Grey wolf groups gradually approach and surround their prey through several formulas:

(5) $$D=\left|C\cdot X_{p}(t)-X(t)\right|$$

(6) $$X(t+1)=X_{p}(t)-A\cdot D$$

where t represents the number of iterations, X(t) and $X_{p}(t)$ represent the position vector between the wolf and its prey, A and C represent the cooperation coefficient vector, and D is the distance between the individual wolf pack and the target. The formula for calculating coefficient vectors A and C is:

(7) $$A=2f\cdot r_{1}-f$$

(8) $$C=2\cdot r_{2}$$

where, as the number of iterations increases, f decays linearly from 2 to 0. To enable some agents to reach an optimal position, $r_{1}$ and $r_{2}$ take values between [0, 1].

When hunting, GWO thinks that α, β, and δ are better at predicting the location of prey. Therefore, individual grey wolves will judge the distance $D_{\alpha }$, $D_{\beta }$, and $D_{\delta }$ between themselves and α, β, and δ; calculate their moving distances $X_{1}$, $X_{2}$, and $X_{3}$ toward the three, respectively; and finally move within the circle of the three. The moving formula is shown in Equation (9).

(9) $$\left\{ \begin{array}{l} D_{\alpha }=\left|C_{1}\cdot X_{\alpha }-X(t)\right|\\ D_{\beta }=\left|C_{1}\cdot X_{\beta }-X(t)\right|\\ D_{\delta }=\left|C_{1}\cdot X_{\delta }-X(t)\right| \end{array}\right.$$

(10) $$\left\{ \begin{array}{l} X_{1}=X_{\alpha }-A\cdot D_{\alpha }\\ X_{2}=X_{\beta }-A\cdot D_{\beta }\\ X_{3}=X_{\delta }-A\cdot D_{\delta } \end{array}\right.$$

(11) $$X(t+1)=(X_{1}+X_{2}+X_{3})/3$$

### 4.2. Multi-Strategy Improved Grey Wolf Optimization Algorithm

#### 4.2.1. Sobol-Sequence Initialization Population Strategy

In the swarm intelligence algorithm, whether the initial population distribution is uniform will have a great impact on the optimization performance of the algorithm. GWO initializes the population randomly, resulting in the distribution of the initial population being extremely scattered, which will have a great impact on the algorithm’s solving speed and optimization accuracy. Therefore, this paper initializes population individuals through the Sobol sequence. The Sobol sequence is a kind of low difference sequence [27], which is based on the smallest prime number, two. To produce a random sequence X∈[0,1], an irreducible polynomial of the highest order k in base two is first required to produce a set of predetermined directional numbers $V=[V_{1},V_{2},\cdots ,V_{k}]$, and then the index value of the binary sequence $i={\left(\cdots i_{3}i_{2}i_{1}\right)}_{2}$ is required; then, the nth random number generated by the Sobol sequence is:

(12) $$X_{i}=i_{1}V_{1}⊕i_{2}V_{2}⊕\cdots \;\;i={\left(\cdots i_{3}i_{2}i_{1}\right)}_{2}$$

**Figure 2:**
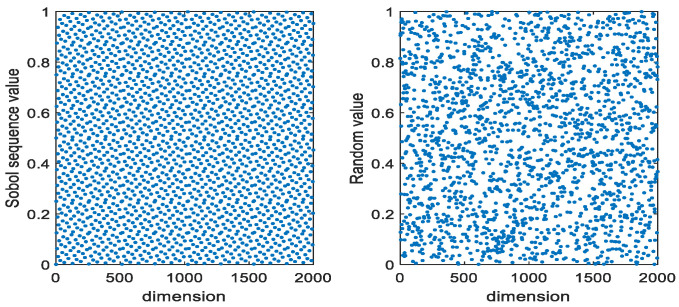
The Sobol sequence and random method to generate individual distribution maps.

The distribution of individuals with the same population size in the same dimensional space is shown in Figure 2. From Figure 2, it can be seen that the distribution of the population initialized using the Sobol sequence is more uniform than that generated randomly, which enables the population to traverse the entire search space better.

#### 4.2.2. Exponential Rule Convergence-Factor Adjustment Strategy

The parameter A is an important parameter regulating global exploration and local development in GWO, which is mainly affected by convergence factor f. In GWO, when |A|>1, the grey wolf population searches the entire search domain for potential prey, and when |A|≤1, the grey wolf population will gradually surround and capture prey.

In GWO, the value of convergence factor f decreases linearly from 2 to 0 with the increase in the number of iterations, which cannot accurately reflect the complex random search process in the actual optimization process. In addition, in the process of algorithm iteration, the same method was used to calculate the enveloping step length for grey wolf individuals with different fitness, which did not reflect the differences among individual grey wolves. Therefore, this paper introduces an updated mode of convergence factor based on exponential rule changes, whose equation is as follows:

(13) $$f^{\prime }=2e^{-t/T}$$

**Figure 3:**
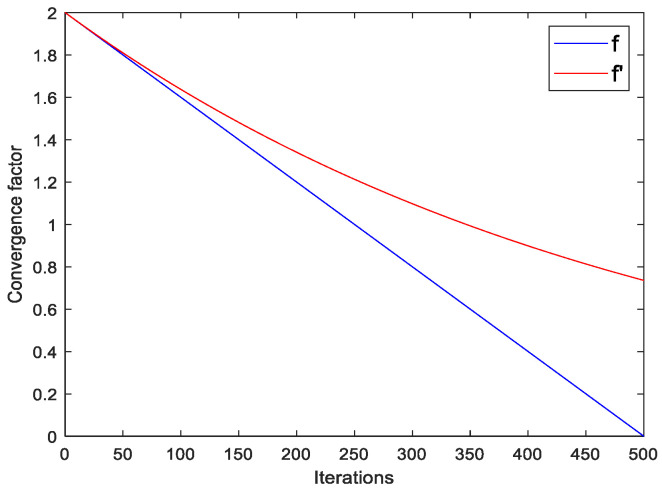
The convergence factor comparison curve.

The curves of the linear convergence factor and exponential regular convergence factor proposed in this paper with the number of iterations are shown in Figure 3. As can be seen from Figure 3, the convergence factor f in GWO decreases linearly with the increase in iterations, resulting in incomplete prey searches in the early stage and slow convergence in the later hunting process. The convergence factor $f^{\prime }$, which varies exponentially, can thoroughly search for prey in the early stages of the algorithm, thereby enhancing its global optimization performance.

#### 4.2.3. Adaptive Location-Update Strategy

In GWO, the initializing α, β, and δ solutions are recorded and retained until they are replaced by a better-fitting individual in the iterative process. In other words, if there is no better α, β, and δ solution in the population than that recorded in the t generation, the new population will still update its position toward wolves α, β, and δ. But when these three are in the local optimal area, then the whole population cannot obtain the optimal solution. Moreover, the average value of $X_{1}$, $X_{2}$, and $X_{3}$ in GWO cannot show the importance of α, β, and δ. Therefore, a new adaptive location-update strategy is proposed, which is expressed as follows:

(14) $$\left\{ \begin{array}{l} W_{1}=\frac{\left|X_{1}\right|}{\left|X_{1}\right|+\left|X_{2}\right|+\left|X_{3}\right|+\varepsilon }\\ W_{2}=\frac{\left|X_{2}\right|}{\left|X_{1}\right|+\left|X_{2}\right|+\left|X_{3}\right|+\varepsilon }\\ W_{3}=\frac{\left|X_{3}\right|}{\left|X_{1}\right|+\left|X_{2}\right|+\left|X_{3}\right|+\varepsilon } \end{array}\right.$$

(15) $$g=\frac{T-t}{T}(g_{initial}-g_{final})+g_{final}$$

where g is the inertia weight. The mathematical expression of grey wolf position update is shown in Equation (16).

(16) $$X(t+1)=\frac{W_{1}X_{1}+W_{2}X_{2}+W_{3}X_{3}}{3}g+X_{1}\frac{t}{T}$$

#### 4.2.4. Cauchy–Gaussian Hybrid Mutation Strategy

In order to avoid the local optimization of the basic GWO algorithm, this paper introduces the Cauchy–Gaussian hybrid mutation strategy combining Cauchy and Gaussian distribution, and gives the best individuals the Cauchy–Gaussian perturbation. The Cauchy–Gaussian operator can generate a large step length to avoid the algorithm falling into local optimality, and its expression is as follows:

(17) $$X_{new}^{*}(t)=X^{*}(t)\cdot (1+\lambda_{1}cauchy(0,1)+\lambda_{2}Gauss(0,1))$$

(18) $$\lambda_{1}=1-\frac{t^{2}}{T_{\max}^{2}}$$

(19) $$\lambda_{2}=\frac{t^{2}}{T_{\max}^{2}}$$

where $X_{new}^{*}(t)$ is the value obtained using Cauchy–Gaussian perturbation, cauchy(0,1) is the Cauchy operator, and Gauss(0,1) is the Gaussian operator.

**Figure 4:**
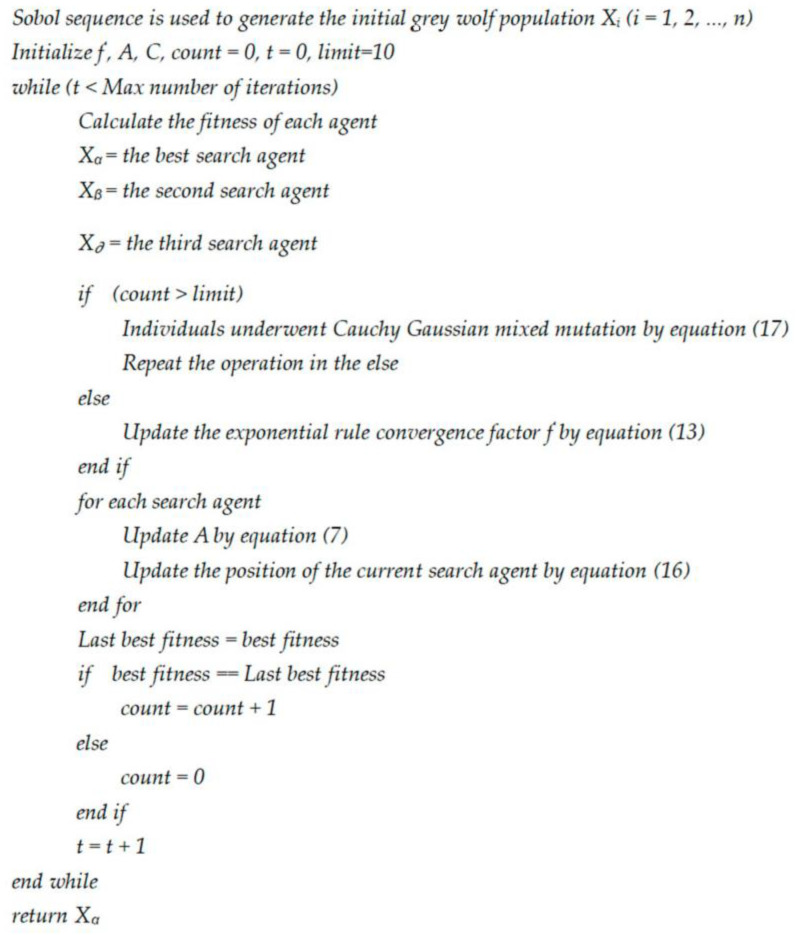
The pseudocode of MSGWO.

The pseudocode of MSGWO is shown in Figure 4.

### 4.3. Improved Performance Test of Grey Wolf Optimization Algorithm

CEC23 sets of commonly used test functions are important examples of testing algorithm performance [28]. In an effort to test the performance of the MSGWO raised in this article, fifteen test functions in the CEC23 group of commonly used test functions were selected for verification, in which F_1_ to F_7_ were single-peak benchmark functions, F_8_ to F_13_ were multi-peak benchmark functions, and F_14_ to F_15_ were fixed-dimensional multi-peak test functions. The computing platform performance was based on IntelI CITM) i5-6500 CPU, 3.20 GHz main frequency, and 8 GB memory. The details of the test function are shown in Table 1.

#### 4.3.1. Comparison Experiment between MSGWO and Standard Optimization Algorithm

In an effort to objectively verify the performance of MSGWO, the population size was set to 30 times, the maximum number of iterations was set to 500 times, and each algorithm was run independently 30 times. Algorithms to be compared in the experiment included the bat optimization algorithm (BOA) [29], whale optimization algorithm (WOA) [30], grey wolf optimization algorithm (GWO), gravity search algorithm (GSA) [31], particle swarm optimization algorithm (PSO) [32], and artificial bee colony algorithm (ABC) [33]. The parameters of all the comparison algorithms in the experiment were the same as those recommended in the original literature. The mean value and standard deviation of the optimal value of the simulation results were taken as the evaluation indexes of the algorithm performance, and the results are shown in Table 2. The test results shown in bold black in Table 2 are the best for comparison.

It can be seen from the data in Table 2 that MSGWO obtained the optimal mean and variance in functions F_1_–F_4_, F_7_, F_9_–F_13_, and F_15_. In the function F_5_, MSGWO obtained the best average value, but its stability was worse than BOA. In the function F_6_, MSGWO obtained the best average value, but its stability was worse than WOA and GWO. In the function F_8_, MSGWO achieved the best average, but its stability was the worst. In the function F_14_, MSGWO obtained the best average value, but its stability was worse than that of the ABC algorithm. It can be seen that MSGWO obtained the optimal average value in all the selected test functions. Although the stability of the algorithm was worse in some individual functions than that of some comparison algorithms, MSGWO still had better optimization performance on the whole.

The simulation results show that MSGWO had better optimization performance under different benchmark test functions. This shows that compared with GWO, MSGWO enhances the local search ability, thus increasing the solution accuracy, and for multi-modal test functions, MSGWO has a strong local optimal avoidance ability, and can better find the optimal solution. When other algorithms have low optimization accuracy or even cannot converge, MSGWO still has high solving accuracy.

**Figure 5:**
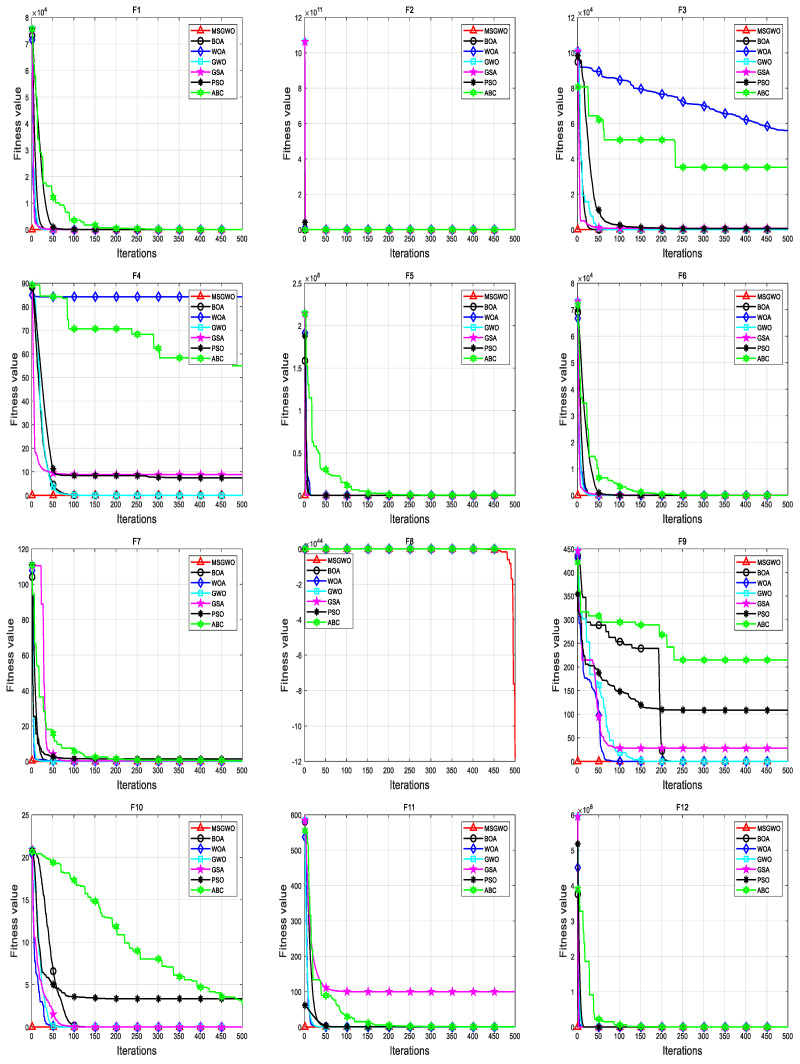

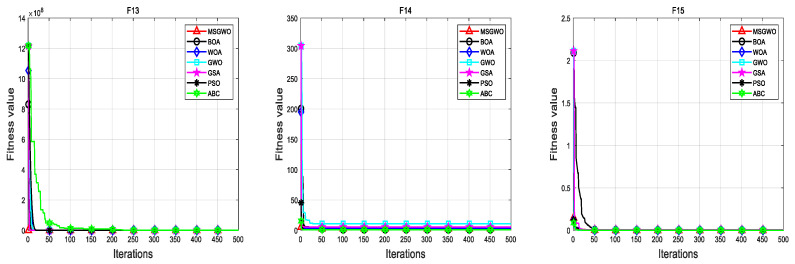
The convergence curve of MSGWO is compared with that of standard algorithm.

In order to explore the influence of improvement strategies on the algorithm convergence speed, the convergence curves of each algorithm under 15 benchmark test functions are shown in Figure 5. As can be seen from Figure 5, MSGWO has high precision and the fastest convergence rate of the optimal solution in the comparison algorithm, which effectively saves the optimization time.

#### 4.3.2. Comparison Experiment between MSGWO and Improved Optimization Algorithm

In an effort to further test the performance of the MSGWO, the population size was set to 30 times, the maximum number of iterations was set to 500 times, and each algorithm was independently run 30 times. Comparative experimental analysis was conducted between MSGWO and GWO, MEGWO [34], mGWO [35], IGWO [36], and MPSO [37]. The mean value and standard deviation of the optimal value of the simulation results were taken as the evaluation indexes of the algorithm performance, and the results are shown in Table 3. The test results shown in bold black in Table 3 are the best for comparison.

It can be seen from the data in Table 3 that for the optimization accuracy of the algorithm, MSGWO obtained the optimal average value in the function F_1_–F_15_. In terms of algorithm stability, the stability of the MSGWO was worse than that of the IGWO algorithm in F_5_; worse than those of the GWO, mGWO, IGWO, and MPSO algorithms in F_6_; the worst in F_8_; worse than those of the mGWO and IGWO algorithms in F_12_; worse than that of the MPSO algorithm in F_14_; and worse than that of MEGWO in F_15_. However, in the other nine test functions, its stability was better than the comparison algorithm, so the overall stability was still the best.

**Figure 6:**
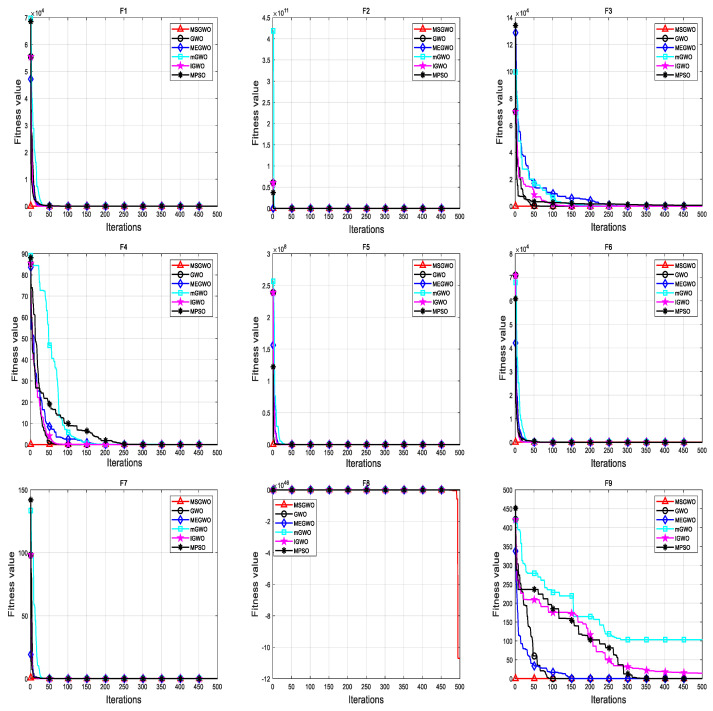

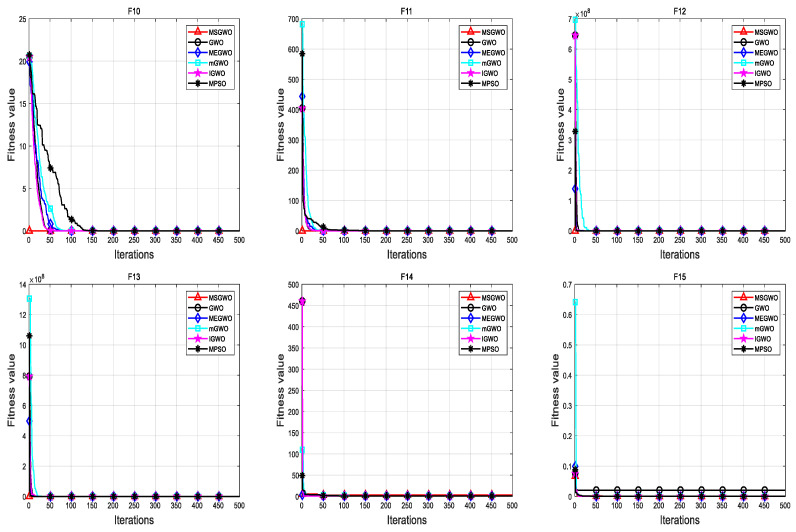
The convergence curves are compared between MSGWO and the improved algorithm.

The convergence curves of the MSGWO algorithm and improved algorithms under 15 benchmark functions are shown in Figure 6. It can be seen from the convergence curves of each test function in Figure 6 that MSGWO has better local extreme value escape ability, overall optimization coordination, and convergence performance than the comparison algorithm.

#### 4.3.3. Wilcoxon Rank Sum Test

In order to verify whether there were significant differences between MSGWO and other comparison algorithms, the Wilcoxon rank sum test was used for statistical analysis of the experimental data. For each test function, the results of 30 independent optimizations of MSGWO were compared with the 30 independent optimizations of the standard optimization algorithms (WOA, GWO, BOA, GSA, PSO, ABC) and improved optimization algorithms (MEGWO, mGWO, IGWO, MPSO) using the Wilcoxon rank sum test at a significance level of 5%. The population size of all algorithms was set to 30, with 500 iterations. The *p* value of the test result was less than 0.05, indicating that there were significant differences between the comparison algorithms. The symbols “+”, “−”, and “=“ of R indicate that the performance of MSGWO was better than, worse than, and equivalent to the comparison algorithm, respectively, and N/A indicates that a significance judgment could not be made. The test results are shown in Table 4 and Table 5, respectively.

As can be seen from Table 4, comparing the optimization results of MSGWO with those of WOA, GWO, BOA, GSA, PSO, and ABC on 15 test functions, the *p* values of the test results are all less than 0.05, and the R values are all +, indicating that the optimization results of MSGWO are significantly different from those of other six algorithms. Additionally, MSGWO is significantly better, which shows the superiority of the MSGWO algorithm statistically.

As can be seen from Table 5, compared with the optimization results of the five improved algorithms on 15 test functions, the *p* values of the test results of MSGWO are all less than 0.05, and R is +/=, which indicates that the optimization results of MSGWO are significantly different from the optimization results of the five improved algorithms, and MSGWO is significantly better. This result shows the superiority of the MSGWO algorithm statistically.

#### 4.3.4. Population Diversity Analysis of MSGWO

In an effort to further illustrate the effectiveness of the proposed algorithm, the diversity of population particles during evolution was analyzed. Population diversity measurements can accurately evaluate whether a population is being explored or exploited [38], and the specific calculation formula is as follows:

(20) $$I_{C}(t)=\sqrt{\sum_{i=1}^{N}\sum_{d=1}^{D}{\left(x_{id}(t)-c_{d}(t)\right)}^{2}}$$

(21) $$c_{d}(t)=\frac{1}{D}\sum_{i=1}^{N}x_{id}(t)$$

where $I_{C}$ represents the dispersion between the population and the center of mass $c_{d}$ in each iteration, and $x_{id}$ represents the value of the d dimension of the ith individual at the time of iteration t.

**Figure 7:**
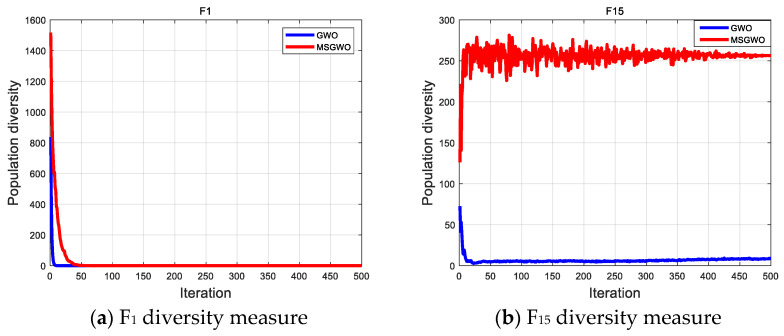
Population diversity measurement analysis.

A small population diversity measure indicates that particles converge near the population center, that is, develop in a small space. A large population diversity measure indicates that the particles are far from the center of the population, that is, they explore in a larger space. Unimodal function F_1_ and multi-modal function F_15_ of the commonly used test functions of CEC23 were selected as representatives to analyze the population diversity measurements of MSGWO and GWO, respectively. The experimental results are shown in Figure 7a,b.

As can be seen from Figure 7, the population diversity measure of the GWO algorithm decreased at the fastest speed in F_1_ and F_15_, which is not conducive to sufficient space exploration in the early stage and is easy to fall into local optimization. In F_1_, the MSGWO algorithm maintained a high level of population diversity in the early stage of evolution, fully satisfying the exploration of particles in the whole space, while the population diversity decreased rapidly in the middle and late stages of evolution, indicating that the algorithm has a good development ability. In F_15_, MSGWO population diversity fluctuated greatly and remained at a high level, indicating that the algorithm has a good global exploration ability.

## 5. Bearing Fault Detection

### 5.1. Parameter Adaptive Multistable Stochastic Resonance Strategy

In SR performance measurement indicators, signal-to-noise ratio (SNR) is commonly used and plays an important role. In this paper, the SNR is used as the target of optimization, that is, the fitness function. The formula for calculating the SNR is as follows [39]:

(22) $$\mathrm{SNR}=10\log_{10}\frac{A_{t}}{\sum_{n=0}^{N/2}A_{n}}$$

where $A_{t}$ is the amplitude of the target frequency, $A_{n}$ is the amplitude of frequencies other than the target frequency in the input signal, and N is the number of samples.

**Figure 8:**
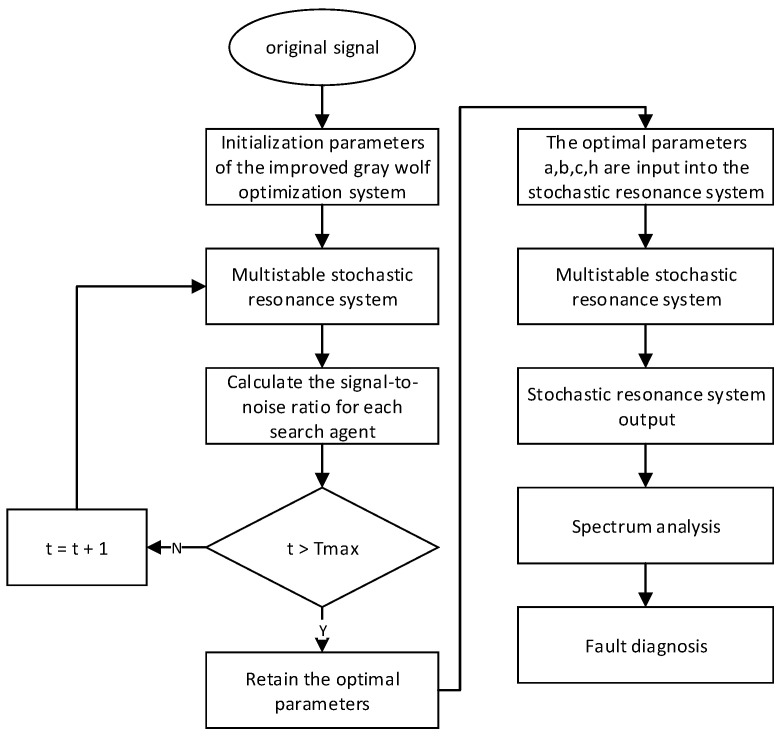
The flow diagram of the proposed algorithm.

Based on the above analysis, the flow chart of the bearing fault-detection method proposed in this paper is shown in Figure 8, and its specific steps are as follows:

Step 1: Input noisy signals and initialize MSGWO parameters. The range of a is [0, 0.5]; the range of b, c, and h are [0, 10]. The maximum number of iterations is 200 and the number of grey wolf populations is 30.

Step 2: Run the MSGWO, calculate the SNR according to Equation (22), then update the individual position, iterate to the maximum number of iterations, and finally terminate the iteration.

Step 3: Substitute the optimal solutions of a, b, c, and h into the SR system for operation, and subject the output of the SR system to fast Fourier transform to obtain the frequency domain. Then, analyze the output of the SR according to the frequency domain, and capture the fault frequency.

### 5.2. CWRU Bearing Data Set

**Figure 9:**
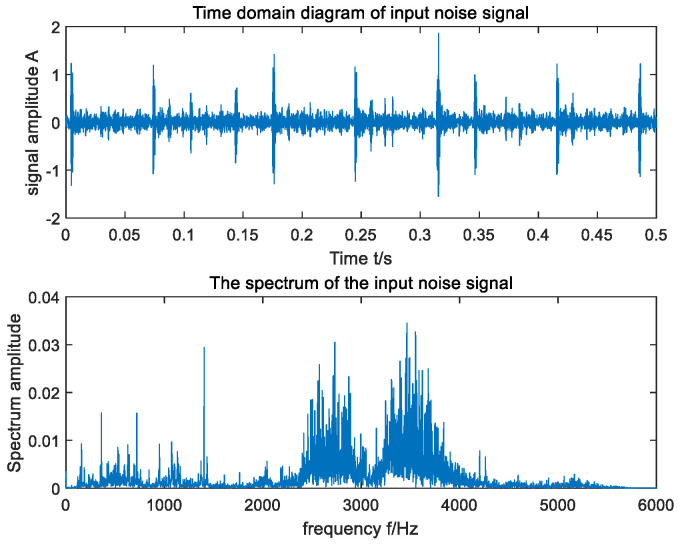
Time domain waveform and FFT spectrum of CWRU input signal.

**Figure 10:**
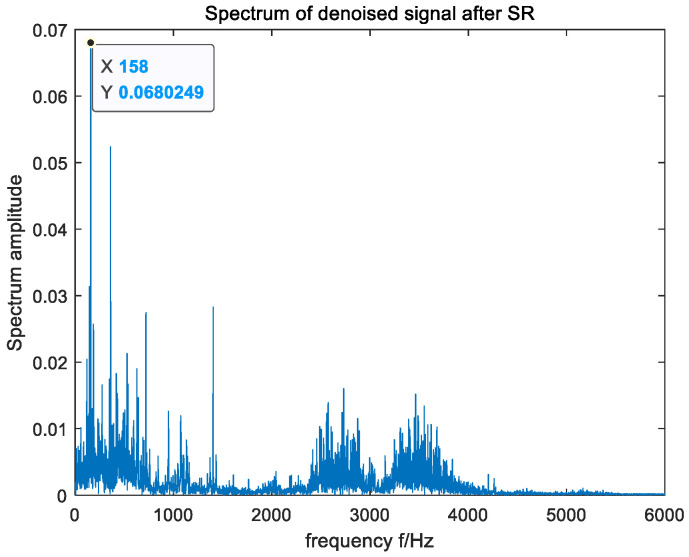
The FFT spectrum of the output signal processed by the raised algorithm.

In an effort to verify the applicability of the raised method in actual fault-signal detection, the open bearing-fault data set of CWRU was selected for the experiment [40], and the driving end bearing model 6205-2RS was used. Since the rotating speed of the bearing was 1750 rpm, the fault characteristic frequency of the inner ring was calculated to be 158 Hz. In the experiment, the sampling frequency was set to 12 kHz, and the data length of the signal was 12,000. The time domain and frequency domain waveforms of the input signal are shown in Figure 9, and the output signal-to-noise ratio was SNR=−37.77. As can be seen from Figure 9, the fault frequency of the original signal was difficult to capture in its frequency domain due to the influence of environmental noise. In order to ensure the accuracy of the experimental results, the average method of 30 experiments was adopted. The optimal parameters optimized by MSGWO were as follows: a=0.033, b=0.567, c=0.082, and h=0.086. We substituted the four parameters a, b, c, and h into the SR system to obtain the frequency domain waveform of its output, as shown in Figure 10. The output signal-to-noise ratio was SNR=−26.92, which was 10.85 dB higher than that of the input. According to the frequency domain waveform diagram in Figure 10, it can be observed that there was a clear spike at the target frequency, and the amplitude of the peak frequency was much larger than the amplitude of other surrounding frequencies. It can be seen that the method in this paper can effectively detect the bearing fault signal.

In the case of the same parameters, the raised method was compared with five bearing fault-detection methods based on the improved algorithms to optimize the SR parameters. In an effort to ensure the accuracy of the experimental results, the method of averaging 30 experiments was adopted. The comparison experiment results are shown in Table 6. The test results shown in black bold in Table 6 are the best results for comparison.

According to the data in Table 6, compared with five bearing fault-detection methods based on improved algorithms to optimize SR parameters, the raised method had the highest SNR, but the convergence speed was slower than that of bearing fault-detection methods based on IGWO and MPSO. Since the SNR was taken as the evaluation index in bearing fault detection, the proposed method had some advantages over the five bearing fault-detection methods based on the improved algorithm to optimize the SR parameters.

### 5.3. MFPT Bearing Data Set

**Figure 11:**
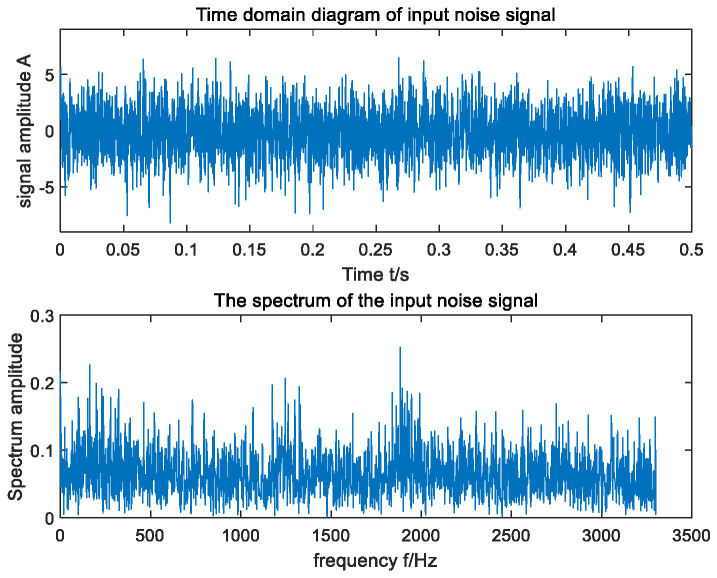
Time domain waveform and FFT spectrum of MFPT input signal.

**Figure 12:**
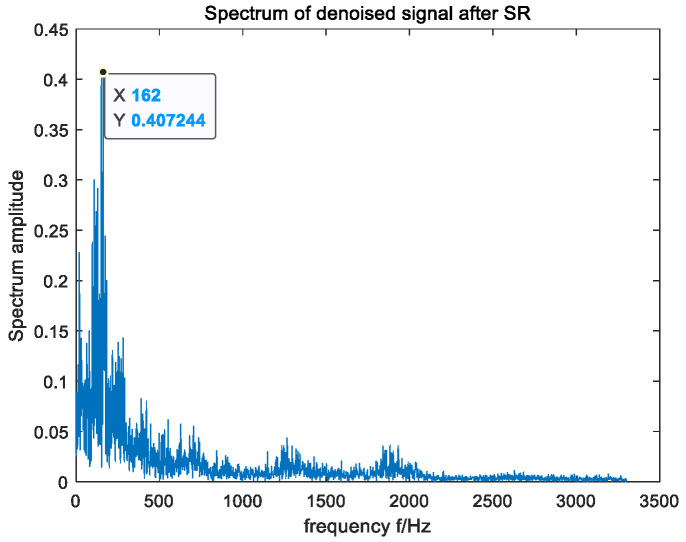
The FFT spectrum of the output signal processed by the proposed algorithm.

In an effort to further verify the applicability of the raised method in actual fault-signal detection, the bearing data set of the MFPT in the United States was selected as the research object [41] to detect the outer-ring signal of the faulty bearing. The input shaft speed of the selected outer ring fault signal was 25 Hz, the load was 25, and the fault characteristic frequency was calculated to be 162 Hz. The time domain and frequency domain waveform of the input signal are shown in Figure 11. According to Figure 11, due to the influence of ambient noise, the fault frequency of the original signal was submerged in the noise and was difficult to be captured in its frequency domain. In an effort to ensure the accuracy of the experimental results, the average method of 30 experiments was adopted. The optimal parameters optimized by MSGWO were as follows: a=0.500, b=9.571, c=0.019, and h=0.409. We substituted the four parameters a, b, c, and h into the SR system to obtain the frequency domain waveform of its output, as shown in Figure 12. According to the frequency domain waveform diagram in Figure 12, it can be observed that the amplitude of the target frequency was the largest in its frequency domain and was much larger than the amplitude of other surrounding frequencies. This further proves that the raised method can detect the bearing fault signal effectively.

In the case of the same parameters, the raised method was compared with five bearing fault-detection methods based on the improved algorithms to optimize the SR parameters. In an effort to ensure the accuracy of the experimental results, the method of averaging 30 experiments was adopted. The comparison experiment results are shown in Table 7. The test results shown in black bold in Table 7 are the best results for comparison.

According to the data in Table 7, compared with five bearing fault-detection methods based on the improved algorithms to optimize SR parameters, the method raised in this article had a larger SNR and better time performance. Therefore, the method proposed in this article has certain advantages over the comparative method.

### 5.4. Bearing-Fault Diagnosis of Crystal Growing Furnace

**Figure 13:**
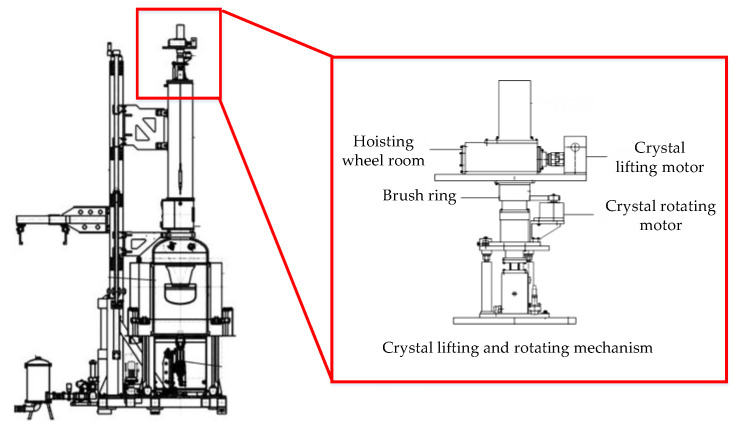
Crystal growing furnace and crystal lifting and rotating mechanism.

**Figure 14:**
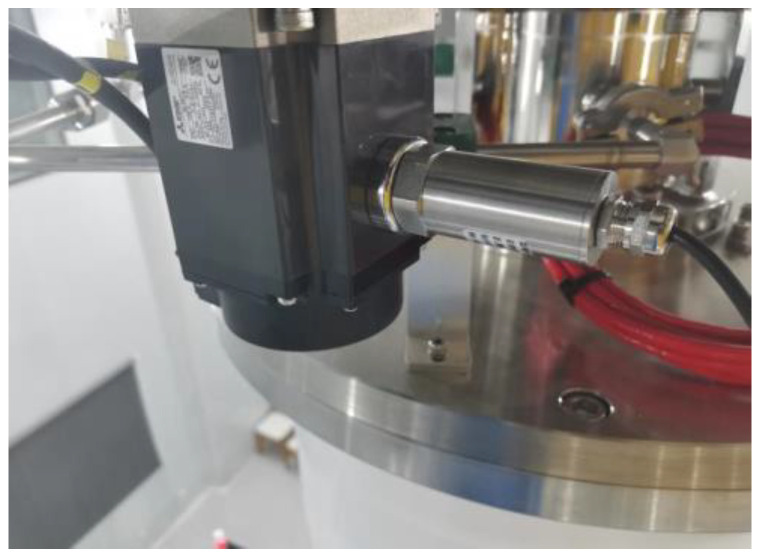
Vibration sensor installation position.

In this paper, the crystal lifting and rotating mechanism of a crystal growing furnace was taken as the actual test object, as shown in Figure 13. The crystal growing furnace is the major equipment for producing wafers. The mechanism is composed of two Mitsubishi HG-KR73 servo motors, the crystal lift motor is used to lift the crystal upward, and the crystal rotating motor is used to drive the crystal to spin during the growth process. Because the stability of crystal rotating is an important factor to determine the crystal formation and crystal quality, it is necessary to accurately monitor the fault of the crystal rotating motor. The experiment object was the motor of a certain type of electronic-grade silicon single-crystal growing furnace. The purpose was to detect the failure frequency of the crystal rotating motor. A certain type of three-dimensional vibration sensor was used in the experiment, and its connection with the motor is shown in Figure 14. As shown in Figure 14, the vibration sensor was adsorbed on the motor, and information such as vibration displacement, vibration speed, and vibration frequency can be collected. The deceleration ratio of the crystal rotating system was 100:1, that is, when the crystal rotating speed was 10 rad/min, the speed of the crystal rotating motor was 1000 rad/min.

**Figure 15:**
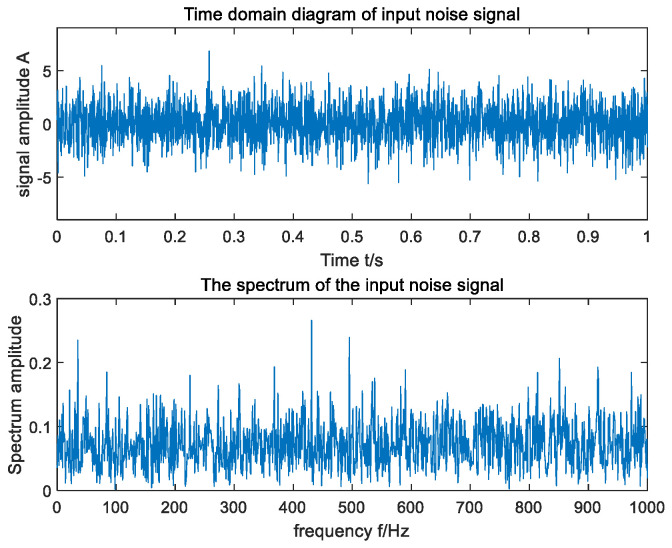
Original vibration signal of crystal rotating motor.

**Figure 16:**
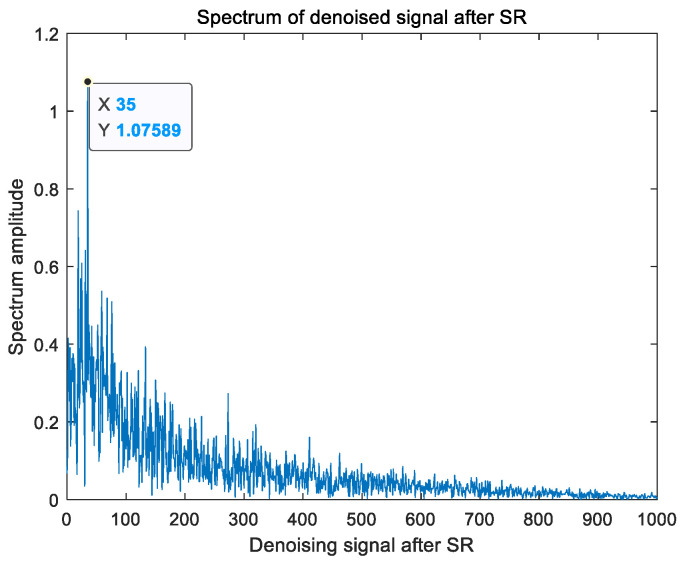
Spectrum amplitude of motor fault.

The vibration signal of the motor collected by the vibration sensor is shown in Figure 15. As can be seen from Figure 15, the time domain signal of the actual motor fault collected by the vibration sensor is very weak, completely submerged in the noise, and the frequency domain signal cannot distinguish the fault frequency. The method proposed in this paper was used to detect the fault frequency of the crystal rotating motor, and the test results are shown in Figure 16. It can be seen from Figure 16 that the algorithm increased the frequency domain amplitude of the fault signal and effectively detected that the fault frequency of the crystal motor was 35 Hz.

## 6. Conclusions

Taking bearing fault-signal detection as the research object, this paper proposes a bearing fault-detection method based on an improved grey wolf algorithm to optimize multistable stochastic resonance parameters, aiming at the problems that multistable stochastic resonance system parameters are difficult to select and basic grey wolf optimization algorithm is prone to local optimization and low convergence accuracy. This method improved the grey wolf optimization algorithm. Firstly, the Sobol sequence was used to initialize the grey wolf population to improve the diversity of the population. Secondly, the exponential rule convergence factor was used to balance the global search and local development stages of the algorithm. At the same time, the adaptive position-update strategy was introduced to improve the accuracy of the algorithm. Additionally, we used Cauchy–Gaussian hybrid variation to improve the ability of the algorithm to escape from the local optimal area. The performance of the proposed algorithm was verified using experiments with 15 benchmark test functions in the CEC23 group of common test functions. The results show that the multi-strategy improved grey wolf optimization algorithm has better optimization performance. Then, the improved grey wolf optimization algorithm was used to optimize the parameters of the multistable stochastic resonance algorithm, so as to realize the detection of bearing fault signals. Finally, the bearing data sets of Case Western Reserve University and the Association for Mechanical Fault Prevention Technology were analyzed and diagnosed with the proposed bearing fault-detection method, and the optimization results were compared with other improved algorithms. At the same time, the method proposed in this paper was used to diagnose the fault of the bearing of the lifting device of a single-crystal furnace. The experimental results show that this method can be used to detect the bearing fault signal and can effectively enhance the fault signal in the noise. Compared with other optimized bearing fault-detection methods based on improved intelligent algorithms, the proposed method has the advantages of fast convergence, high parameter optimization accuracy, and strong robustness.

In the future, this paper will study the following two aspects: Firstly, the MSGWO needs to be further improved to improve its stability due to its poor stability in individual test functions. Secondly, the bearing fault-detection method proposed in this paper will be applied to the bearing fault detection of rotating machinery in different industries, and the corresponding improvement will be made according to the actual detection results, so as to improve the applicability of the bearing fault-detection method proposed in this paper to different industries.

## Figures and Tables

**Table 1:** Benchmark functions.

Function	Dim	Range	Optima
$F_{1}(x)=\sum_{i=1}^{n}x_{i}^{2}$	30	[−100, 100]	0
$F_{2}(x)=\sum_{i=1}^{n}\left|x_{i}\right|+\prod_{i=1}^{n}\left|x_{i}\right|$	30	[−10, 10]	0
$F_{3}(x)={\sum_{i=1}^{n}\left(\sum_{j-1}^{i}x_{j}\right)}^{2}$	30	[−100, 100]	0
$F_{4}(x)=\max_{i}\left\{\left|x_{i}\right|,1\leq i\leq n\right\}$	30	[−100, 100]	0
$F_{5}(x)=\sum_{i=1}^{n-1}\left[100{\left(x_{i+1}-x_{i}^{2}\right)}^{2}+{\left(x_{i}-1\right)}^{2}\right]$	30	[−30, 30]	0
$F_{6}(x)={\sum_{i=1}^{n}\left(\left[x_{i}+0.5\right]\right)}^{2}$	30	[−100, 100]	0
$F_{7}(x)=\sum_{i=1}^{n}ix_{i}^{4}+random[0,1)$	30	[−1.28, 1.28]	0
$F_{8}(x)=\sum_{i=1}^{n}-x_{i}\sin(\sqrt{\left|x_{i}\right|})$	30	[−500, 500]	−418.98 × Dim^n^
$F_{9}(x)=\sum_{i=1}^{n}\left[x_{i}^{2}-10\cos(2\pi x_{i})+10\right]$	30	[−5.12, 5.12]	0
$F_{10}(x)=-20\exp\left(-0.2\sqrt{\frac{1}{n}\sum_{i=1}^{n}x_{i}^{2}}\right)-\exp\left(\frac{1}{n}\sum_{i=1}^{n}\cos\left(2\pi x_{i}\right)\right)+20+e$	30	[−32, 32]	0
$F_{11}(x)=\frac{1}{4000}\sum_{i=1}^{n}x_{i}^{2}-\prod_{i=1}^{n}\cos\left(\frac{x_{i}}{\sqrt{i}}\right)+1$	30	[−600, 600]	0
$\begin{array}{l} F_{12}(x)=\frac{\pi }{n}\left\{10\sin(\pi y_{1})+\sum_{i=1}^{n-1}{\left(y_{i}-1\right)}^{2}\left[1+10\sin^{2}(\pi y_{i+1})\right]+{\left(y_{n}-1\right)}^{2}\right\}\\ +\sum_{i=1}^{n}u(x_{i},10,100,4)\\ y_{i}=1+\frac{x_{i}+1}{4}\\ u(x_{i},a,k,m)=\left\{ \begin{array}{l} k{\left(x_{i}-a\right)}^{m}\;x_{i}>a\\ 0\;\;\;\;-a<x_{i}<a\\ k{\left(-x_{i}-a\right)}^{m}\;\;x_{i}<-a \end{array}\right. \end{array}$	30	[−50, 50]	0
$\begin{array}{l} F_{13}(x)=0.1\left\{\sin^{2}(3\pi x_{1})+\sum_{i=1}^{n}{\left(x_{i}-1\right)}^{2}[1+\sin^{2}(3\pi x_{i}+1)]+{\left(x_{n}-1\right)}^{2}[1+\sin^{2}(2\pi x_{n})]\right\}\\ +\sum_{i=1}^{n}u(x_{i},5,100,4) \end{array}$	30	[−50, 50]	0
$F_{14}(x)={\left(\frac{1}{500}+\sum_{j=1}^{25}\frac{1}{j+\sum_{i=1}^{2}{\left(x_{i}-a_{ij}\right)}^{6}}\right)}^{-1}$	2	[−65, 65]	1
$F_{15}(x)={\sum_{i=1}^{11}\left[a_{i}-\frac{x_{1}(b_{i}^{2}+b_{1}x_{2})}{b_{i}^{2}+b_{1}x_{3}+x_{4}}\right]}^{2}$	4	[−5, 5]	0.1484

**Table 2:** The compared results of MSGWO and standard optimization algorithms.

F	Index	WOA	GWO	BOA	GSA	PSO	ABC	MSGWO
F_1_	mean	4.97 × 10^−74^	1.04 × 10^−27^	4.06 × 10^−4^	98.91	11.65	3.54	**0**
	std	2.49 × 10^−73^	1.37 × 10^−27^	8.97 × 10^−5^	106.42	5.28	1.26	**0**
F_2_	mean	2.46 × 10^−52^	9.51 × 10^−17^	4.54 × 10^−9^	4.46	11.69	0.16	**0**
	std	5.61 × 10^−52^	7.47 × 10^−17^	1.26 × 10^−9^	4.47	3.64	0.05	**0**
F_3_	mean	3.87 × 10^4^	3.15 × 10^−5^	1.25 × 10^−11^	1.31 × 10^3^	7.25 × 10^2^	3.37 × 10^4^	**0**
	std	1.48 × 10^4^	9.68 × 10^−5^	8.97 × 10^−13^	4.14 × 10^2^	5.27 × 10^2^	5.45 × 10^3^	**0**
F_4_	mean	59.16	7.78 × 10^−7^	6.15 × 10^−9^	10.07	6.73	51.08	**0**
	std	23.48	8.85 × 10^−7^	4.28 × 10^−10^	1.71	1.26	5.48	**0**
F_5_	mean	27.90	28.44	28.94	3.26 × 10^2^	1.87 × 10^3^	1.40 × 10^5^	**27.08**
	std	0.48	0.82	**0.03**	2.51 × 10^2^	1.15 × 10^3^	6.78 × 10^4^	0.42
F_6_	mean	0.42	0.90	5.75	52.25	9.89	3.96	**0.35**
	std	0.48	**0.38**	0.72	60.45	3.57	0.98	0.54
F_7_	mean	2.54 × 10^3^	2.07 × 10^3^	1.39 × 10^3^	1.36	0.68	0.25	**6.58 × 10^−5^**
	std	2.30 × 10^−3^	7.10 × 10^−4^	7.65 × 10^−4^	2.63	0.33	0.08	**6.62 × 10^−5^**
F_8_	mean	−1.04 × 10^4^	−5.70 × 10^3^	−3.77 × 10^4^	−2.48 × 10^3^	−2.22 × 10^3^	−4.98 × 10^3^	**−5.47 × 10^58^**
	std	1.73 × 10^3^	1.18 × 10^3^	3.80 × 10^2^	5.29 × 10^2^	5.89 × 10^2^	**3.55 × 10^2^**	1.81 × 10^59^
F_9_	mean	0.15	3.63	6.72	38.94	92.15	2.33 × 10^2^	**0**
	std	0.83	4.07	36.10	10.12	16.83	15.05	**0**
F_10_	mean	5.51 × 10^−15^	1.03 × 10^−13^	5.81 × 10^−9^	0.55	5.43	1.89	**8.88 × 10^−16^**
	std	2.77 × 10^−15^	2.23 × 10^−14^	7.12 × 10^−10^	0.61	1.18	0.57	**0**
F_11_	mean	0.03	3.02 × 10^−3^	5.22 × 10^−12^	1.01 × 10^3^	0.45	1.02	**0**
	std	0.09	5.70 × 10^−3^	2.40 × 10^−12^	11.85	0.12	0.03	**0**
F_12_	mean	0.05	0.07	0.66	3.12	4.40	17.54	**0.05**
	std	0.13	0.27	0.16	1.10	1.98	8.64	**0.10**
F_13_	mean	0.51	0.71	2.91	27.43	22.29	1.49 × 10^4^	**0.43**
	std	0.29	0.24	0.18	10.75	16.15	2.36 × 10^4^	**0.14**
F_14_	mean	2.90	4.53	1.68	6.66	2.05	1.69	**1.55**
	std	3.20	4.03	0.94	4.61	1.63	**0**	0.70
F_15_	mean	6.13 × 10^−4^	2.47 × 10^−3^	4.39 × 10^−4^	1.17 × 10^−2^	6.15 × 10^−4^	7.04 × 10^−4^	**3.46 × 10^−4^**
	std	3.04 × 10^−4^	6.00 × 10^−3^	1.73 × 10^−4^	6.30 × 10^−3^	4.65 × 10^−4^	5.80 × 10^−4^	**1.69 × 10^−4^**

**Table 3:** Comparison of experimental results between MSGWO and improved algorithms.

F	Index	GWO	MEGWO	mGWO	IGWO	MPSO	MSGWO
F_1_	mean	1.04 × 10^−27^	4.30 × 10^−64^	1.04 × 10^−18^	1.33 × 10^−209^	2.61 × 10^−26^	**0**
	std	1.37 × 10^−27^	2.09 × 10^−63^	2.97 × 10^−18^	0	1.12 × 10^−25^	**0**
F_2_	mean	9.50 × 10^−17^	1.70 × 10^−43^	2.65 × 10^−12^	6.12 × 10^−21^	1.40 × 10^−16^	**0**
	std	6.40 × 10^−17^	5.77 × 10^−43^	1.99 × 10^−12^	6.67 × 10^−21^	2.86 × 10^−16^	**0**
F_3_	mean	3.15 × 10^−5^	0.23	0.68	2.73 × 10^−5^	9.63 × 10^2^	**0**
	std	9.68 × 10^−5^	0.48	0.81	9.57 × 10^−5^	4.81 × 10^2^	**0**
F_4_	mean	7.78 × 10^−7^	2.06 × 10^−5^	0.68	2.93 × 10^−7^	2.05 × 10^−10^	**0**
	std	8.85 × 10^−7^	5.68 × 10^−5^	0.85	1.78 × 10^−7^	4.81 × 10^−10^	**0**
F_5_	mean	28.44	27.94	27.92	27.64	88.91	**27.08**
	std	0.82	9.97	0.58	**0.32**	1.89 × 10^2^	0.42
F_6_	mean	0.90	0.49	0.41	0.43	0.41	**0.36**
	std	0.38	1.14	0.25	**0.19**	0.22	0.54
F_7_	mean	2.07 × 10^−3^	1.01 × 10^−3^	4.68 × 10^−3^	2.80 × 10^−3^	1.68 × 10^−3^	**6.58 × 10^−5^**
	std	7.10 × 10^−4^	9.10 × 10^−4^	1.90 × 10^−3^	1.10 × 10^−3^	8.87 × 10^−4^	**6.62 × 10^−5^**
F_8_	mean	−5.70 × 10^3^	−1.26 × 10^4^	−5.33 × 10^3^	−8.28 × 10^3^	−8.12 × 10^3^	**−5.47** **× 10^58^**
	std	1.18 × 10^3^	**2.15** **× 10^−12^**	1.11 × 10^3^	1.69 × 10^3^	1.12 × 10^3^	1.81 × 10^59^
F_9_	mean	3.63	0	37.94	27.09	23.92	**0**
	std	4.07	0	30.01	22.81	22.64	**0**
F_10_	mean	1.03 × 10^−13^	5.27 × 10^−15^	1.26 × 10^−10^	6.25 × 10^−14^	6.22 × 10^−15^	**8.88** **× 10^−16^**
	std	2.23 × 10^−14^	1.50 × 10^−15^	9.69 × 10^−11^	8.96 × 10^−15^	7.38 × 10^−15^	**0**
F_11_	mean	3.02 × 10^−3^	0	3.83 × 10^−3^	3.37 × 10^−3^	0	**0**
	std	5.70 × 10^−3^	0	9.40 × 10^−3^	6.00 × 10^−3^	0	**0**
F_12_	mean	0.07	0.05	0.05	6.58 × 10^−2^	0.42	**0.05**
	std	0.27	0.56	0.04	**2.00 × 10^−3^**	0.73	0.10
F_13_	mean	0.71	0.46	0.63	0.66	0.45	**0.43**
	std	0.24	0.15	0.22	0.16	0.25	**0.13**
F_14_	mean	4.53	1.78	2.00	1.70	1.99	**1.55**
	std	4.03	2.91	2.76	0.76	**0.36**	0.71
F_15_	mean	2.47 × 10^−3^	3.07 × 10^−4^	1.04 × 10^−3^	8.62 × 10^−4^	5.68 × 10^−4^	**3.46 × 10^−4^**
	std	6.00 × 10^−3^	**3.42 × 10^−15^**	3.60 × 10^−3^	3.00 × 10^−3^	3.36 × 10^−4^	1.69 × 10^−4^

**Table 4:** Wilcoxon rank sum test results for MSGWO and standard algorithms.

F	Index	MSGWO–WOA	MSGWO–GWO	MSGWO–BOA	MSGWO–GSA	MSGWO–PSO	MSGWO–ABC
F_1_	P	1.73 × 10^−6^	1.73 × 10^−6^	1.73 × 10^−6^	1.73 × 10^−6^	1.73 × 10^−6^	1.73 × 10^−6^
	R	+	+	+	+	+	+
F_2_	P	1.73 × 10^−6^	1.73 × 10^−6^	1.73 × 10^−6^	1.73 × 10^−6^	1.73 × 10^−6^	1.73 × 10^−6^
	R	+	+	+	+	+	+
F_3_	P	1.73 × 10^−6^	1.73 × 10^−6^	1.73 × 10^−6^	1.73 × 10^−6^	1.73 × 10^−6^	1.73 × 10^−6^
	R	+	+	+	+	+	+
F_4_	P	1.73 × 10^−6^	1.73 × 10^−6^	1.73 × 10^−6^	1.73 × 10^−6^	1.73 × 10^−6^	1.73 × 10^−6^
	R	+	+	+	+	+	+
F_5_	P	1.06 × 10^−4^	2.88 × 10^−6^	1.73 × 10^−6^	1.92 × 10^−6^	1.73 × 10^−6^	1.73 × 10^−6^
	R	+	+	+	+	+	+
F_6_	P	1.73 × 10^−6^	1.73 × 10^−6^	1.73 × 10^−6^	7.69 × 10^−6^	1.73 × 10^−6^	9.37 × 10^−3^
	R	+	+	+	+	+	+
F_7_	P	2.13 × 10^−6^	1.73 × 10^−6^	1.73 × 10^−6^	1.73 × 10^−6^	1.73 × 10^−6^	1.73 × 10^−6^
	R	+	+	+	+	+	+
F_8_	P	1.73 × 10^−6^	1.73 × 10^−6^	1.73 × 10^−6^	1.73 × 10^−6^	1.73 × 10^−6^	1.73 × 10^−6^
	R	+	+	+	+	+	+
F_9_	P	1.73 × 10^−6^	2.53 × 10^−6^	1.82 × 10^−5^	1.73 × 10^−6^	1.73 × 10^−6^	1.73 × 10^−6^
	R	+	+	+	+	+	+
F_10_	P	2.57 × 10^−6^	1.61 × 10^−6^	1.73 × 10^−6^	1.73 × 10^−6^	1.73 × 10^−6^	1.73 × 10^−6^
	R	+	+	+	+	+	+
F_11_	P	2.57 × 10^−6^	1.73 × 10^−6^	1.73 × 10^−6^	1.73 × 10^−6^	1.73 × 10^−6^	1.73 × 10^−6^
	R	+	+	+	+	+	+
F_12_	P	1.73 × 10^−6^	1.73 × 10^−6^	1.97 × 10^−5^	1.73 × 10^−6^	1.73 × 10^−6^	1.73 × 10^−6^
	R	+	+	+	+	+	+
F_13_	P	1.73 × 10^−6^	1.73 × 10^−6^	0.0021	1.73 × 10^−6^	1.92 × 10^−6^	0.0047
	R	+	+	+	+	+	+
F_14_	P	1.73 × 10^−6^	1.73 × 10^−6^	4.45 × 10^−5^	4.86 × 10^−5^	1.92 × 10^−6^	0.0023
	R	+	+	+	+	+	+
F_15_	P	1.73 × 10^−6^	1.73 × 10^−6^	1.73 × 10^−6^	1.73 × 10^−6^	1.73 × 10^−6^	1.73 × 10^−6^
	R	+	+	+	+	+	+

**Table 5:** Wilcoxon rank sum test results for MSGWO and improved algorithms.

F	Index	MSGWO–GWO	MSGWO–MEGWO	MSGWO–mGWO	MSGWO–IGWO	MSGWO–MPSO
F_1_	P	1.73 × 10^−6^	1.73 × 10^−6^	1.73 × 10^−6^	1.73 × 10^−6^	1.73 × 10^−6^
	R	+	+	+	+	+
F_2_	P	1.73 × 10^−6^	1.73 × 10^−6^	1.73 × 10^−6^	1.73 × 10^−6^	1.73 × 10^−6^
	R	+	+	+	+	+
F_3_	P	1.73 × 10^−6^	1.73 × 10^−6^	1.73 × 10^−6^	1.73 × 10^−6^	1.73 × 10^−6^
	R	+	+	+	+	+
F_4_	P	1.73 × 10^−6^	1.73 × 10^−6^	1.73 × 10^−6^	1.73 × 10^−6^	1.73 × 10^−6^
	R	+	+	+	+	+
F_5_	P	2.88 × 10^−6^	1.73 × 10^−6^	1.73 × 10^−6^	1.73 × 10^−6^	1.92 × 10^−3^
	R	+	+	+	+	+
F_6_	P	1.73 × 10^−6^	1.73 × 10^−6^	1.73 × 10^−6^	9.37 × 10^−3^	1.73 × 10^−6^
	R	+	+	+	+	+
F_7_	P	1.73 × 10^−6^	1.73 × 10^−6^	1.73 × 10^−6^	1.73 × 10^−6^	1.73 × 10^−6^
	R	+	+	+	+	+
F_8_	P	1.73 × 10^−6^	1.73 × 10^−6^	1.73 × 10^−6^	1.73 × 10^−6^	1.73 × 10^−6^
	R	+	+	+	+	+
F_9_	P	2.53 × 10^−6^	0.012	1.73 × 10^−6^	1.73 × 10^−6^	1.73 × 10^−6^
	R	+	=	+	+	+
F_10_	P	1.61 × 10^−6^	3.99 × 10^−7^	1.73 × 10^−6^	1.47 × 10^−6^	1.01 × 10^−7^
	R	+	+	+	+	+
F_11_	P	1.73 × 10^−6^	0.012	1.22 × 10^−4^	7.8 × 10^−3^	0.012
	R	+	=	+	+	=
F_12_	P	1.73 × 10^−6^	0.012	1.22 × 10^−4^	1.73 × 10^−6^	2.9 × 10^−3^
	R	+	=	=	+	+
F_13_	P	1.73 × 10^−6^	1.73 × 10^−6^	1.73 × 10^−6^	1.73 × 10^−6^	1.73 × 10^−6^
	R	+	+	+	+	+
F_14_	P	1.73 × 10^−6^	3.59 × 10^−4^	1.73 × 10^−6^	1.73 × 10^−6^	1.73 × 10^−6^
	R	+	+	+	+	+
F_15_	P	1.73 × 10^−6^	1.73 × 10^−6^	1.7 × 10^−3^	3.11 × 10^−5^	1.73 × 10^−6^
	R	+	+	+	+	+

**Table 6:** Comparison of experimental parameter results based on CWRU dataset.

	GWO	IGWO	MEGWO	mGWO	MPSO	MSGWO
a	0.077	0.080	0.065	0.101	0.055	0.033
b	4.197	6.581	6.305	6.571	8.418	0.567
c	7.206	2.830	6.028	7.417	6.160	0.082
h	0.755	0.888	0.792	0.757	0.763	0.086
Time	15.37	14.24	15.25	15.92	**10.58**	14.72
SNR	−28.35	−28.51	−28.27	−28.37	−28.32	**−** **26.92**

**Table 7:** Comparison of experimental parameter results based on MFPT dataset.

	GWO	IGWO	MEGWO	mGWO	MPSO	MSGWO
a	0.500	0.495	0.500	0.472	0.052	0.500
b	10.00	2.173	8.554	8.247	8.968	9.571
c	0.025	0.488	0.054	3.728	1.287	0.019
h	0.328	0.185	0.257	0.069	0.122	0.409
Time	21.51	25.35	35.52	34.79	22.91	**19.95**
SNR	−26.56	−27.75	−26.82	−27.21	−27.62	**−** **26.42**

## Data Availability

Our source code is available on https://github.com/Zfutur1/Code-information.git.
